# Evaluation of salivary flow in patients following hematopoietic stem cell transplantation: a retrospective study

**DOI:** 10.1016/j.htct.2026.106508

**Published:** 2026-08-06

**Authors:** Beatriz Herchenhorn Chiouhami, Maria Cláudia Rodrigues Moreira, Gabriela de Assis Ramos, Héliton Spíndola Antunes

**Affiliations:** a Instituto Nacional de Cancer, INCA, Rio de Janeiro, RJ, Brazil; b Grupo Oncoclínicas, São Paulo, SP, Brazil

**Keywords:** Hematopoietic stem cell transplantation, Salivary flow, Xerostomia, Chronic GvHD, Oral complications

## Abstract

**Introduction:**

Hematopoietic stem cell transplantation is an effective treatment for hematological diseases but it is frequently associated with oral complications such as hyposalivation and xerostomia, particularly in cases of chronic Graft-versus-Host Disease. The objective of this study was to evaluate unstimulated salivary flow in patients who underwent transplantations at least five years previously and to investigate possible associations between hyposalivation and systemic and oral chronic Graft-versus-Host Disease.

**Methods:**

This retrospective cross-sectional study included 27 patients treated at the National Cancer Institute of Brazil (INCA), with a minimum of five years post-allogeneic transplantation. Clinical data were obtained from medical records. Unstimulated saliva was collected following the protocol described by Davies et al., and salivary flow was classified according to Flink et al. Statistical analysis was performed using SPSS version 18.0 software.

**Results:**

Most patients presented sialometry within normal limits (63.0%), with a median of 0.5 mL/min. Xerostomia was reported by 33.3% of participants. The prevalence of systemic chronic Graft-versus-Host Disease was 81.5%, while that of oral chronic Graft-versus-Host Disease was 63.0%. No statistically significant associations were observed between hyposalivation and systemic or oral chronic Graft-versus-Host Disease.

**Conclusion:**

Although most patients maintain adequate salivary function five years after the transplantation, xerostomia remains a clinically relevant symptom. The absence of statistical association with chronic Graft-versus-Host Disease suggests the need for further studies to explore other contributing factors.

## Introduction

Hematopoietic Stem Cell Transplantation (HSCT) is a therapeutic strategy for the treatment of various malignant and non-malignant hematological diseases, as well as congenital immunological conditions [1].

Among therapeutic approaches for adult malignancies, HSCT stands out due to its association with a high incidence of late adverse effects and long-term complications [2]. Even so, advances in transplantation techniques and clinical support have contributed to an increase in long-term survival rates among HSCT recipients [3].

Despite these advances, patients undergoing HSCT remain susceptible to a wide range of acute and chronic complications. Oral manifestations represent one of the main clinical challenges, with the reported prevalence ranging from 30% to 69% of patients after transplantation [4]. The oral cavity is one of the first regions to reflect the adverse effects of treatment. The main risk factors for the development of oral lesions include the type and source of transplanted stem cells, prior chemotherapy or radiotherapy, conditioning regimen, prophylactic immunosuppression, and the use of multiple medications before and after the procedure [5].

Among the immunological complications associated with allogeneic HSCT, graft-versus-host disease (GvHD) is prominent, being triggered by the activation of donor T cells against genetically distinct recipient tissues. Chronic GvHD (cGvHD) is even more complex, involving autoimmune and alloimmune responses that affect multiple host organs and tissues [6]. Oral manifestations of cGvHD are particularly relevant due to their impact on quality of life and because they represent an important source of morbidity [4,7].

Lymphocytic infiltration of the glands leads to hyposalivation, changes in salivary composition, and the manifestation of sialadenitis [8]. cGvHD itself can promote degenerative changes in the glandular parenchyma, inducing apoptosis, necrosis, and a significant reduction in salivary secretion. Such changes, observed in up to 90% of cases, result in decreased salivary flow, altered pH, and changes in the electrolyte and protein composition of saliva [9,10].

Xerostomia and hyposalivation resulting from salivary gland dysfunction compromise essential functions such as chewing, swallowing, and speaking, and increase the risk of opportunistic infections and rapidly progressing dental caries, thereby negatively impacting quality of life [11]. The implementation of preventive strategies, such as maintaining adequate oral hygiene, using fluoride mouthrinses, and employing salivation-stimulating therapies, can significantly contribute to the management of xerostomia [12]. In cases of marked glandular hypofunction resulting from parenchymal destruction, the use of gel lubricants and specific rinses for oral mucosa lubrication is recommended [13., 14., 15.].

Despite the recognized importance of salivary alterations in post-HSCT patients, studies assessing long-term salivary flow and its association with cGvHD remain scarce, especially five years after the procedure. Therefore, the main objective of the present study was to evaluate the unstimulated salivary flow, through saliva collection and measurement, and the complaint of xerostomia through a subjective assessment, in patients who underwent HSCT at least five years previously. Xerostomia was also evaluated because some patients, despite having normal salivary flow, still report subjective oral dryness. Importantly, the salivary flow rate and xerostomia do not always correlate, highlighting that salivary gland function cannot be fully assessed using objective measures alone. As a secondary objective, the study aimed to investigate the possible association between hyposalivation and the presence of systemic and active oral cGvHD.

## Methods

This cross-sectional, retrospective study included patients enrolled at the Brazilian National Cancer Institute (INCA) who underwent related or unrelated allogeneic HSCT with a minimum follow-up of five years post-transplantation. The study was approved and registered on Plataforma Brasil under protocol number CAAE: 04,066,512.1.1001.5274. All participants provided written informed consent prior to inclusion in the study.

Dental evaluations were performed by the principal investigator on the day patients attended the hospital for consultations or examinations. Patients were assessed before and immediately after HSCT, every three months during the first year, and every six months from the second year onward, or more frequently if oral manifestations of cGvHD or other oral alterations were found.

The clinical evaluation of oral cGvHD followed the National Institutes of Health (NIH) Consensus recommendations [16]. The final severity score (0–12) is the sum of the scores obtained across four oral regions. Individual signs are scored based on clinical presentation: erythema or lichenoid lesions receive 1, 2, or 3 points, while ulcerations receive 3 or 6 points. Based on these findings, disease severity is classified into three categories: Mild (score of 1 for erythema or lichenoid lesions), Moderate (score of 2 for erythema/lichenoid lesions, and/or 3 for ulceration), or Severe (score of 3 for erythema/lichenoid lesions, and/or 6 for ulceration).

Salivary gland biopsies were not performed, since this procedure is not routinely included in the structural evaluation of patients. Unstimulated saliva was collected according to the protocol described by Davies et al. [17]. Patients were evaluated without restrictions on food intake and remained seated upright in a comfortable environment. The initial saliva sample was discarded and subsequent saliva was collected in a decontaminated container and aspirated using a 3 mL syringe. Salivary flow was converted to mL/min and classified according to the criteria described by Flink et al. [18] and Maluf et al. [19] as normal (≥0.2 mL/min), low (0.10–0.19 mL/min), very low (<0.1 mL/min), and asialia (≤0.02 mL/min). Xerostomia was identified based on the patients' subjective complaints of dry mouth. Salivary pH was assessed with a pH 0–14.0 MQuant ® Merck test indicator strip, immediately after saliva collection.

Clinical data were extracted from medical records and recorded in a standardized clinical form within the OpenClinica system. Statistical analyses were conducted using SPSS version 18.0. Continuous variables were described as means ± standard deviation or medians with interquartile range values. Categorical variables were presented as absolute and relative frequencies. Comparisons between categorical variables were performed using the Pearson's chi-square test.

Information on potential confounders, including systemic medication use, smoking habits, and comorbidities, was not available and was not included in the analysis, representing a limitation of the study design.

## Results

The study population consisted of 27 patients, with a median time of 64 months since transplantation. Most patients were male (55.6%), single (51.9%), of mixed race (40.7%), and had a median age of 38 years (Table 1).

**Table 1:** General characteristics of the patients (n = 27).

**Variable**	
Time since transplant (months) - Median (IQR)	64 (62.0 - 69.0)
**Gender** – n (%)	
Female	12 (44.4)
Male	15 (55.6)
**Age (years)** – median (IQR)	38 (15.5 – 49.0)
**Race** – n (%)	
Mixed race	11 (40.7)
White	10 (37.0)
Black	5 (18.5)
Not reported	1 (3.7)

IQR: Interquartile range.

Acute lymphoblastic leukemia (ALL) was the most frequent diagnosis (44.4%). Twenty-one patients (77.8%) received grafts from human leukocyte antigen (HLA)-identical sibling donors, and the most common stem cell source was bone marrow (74.1%). The majority of patients used a conditioning regimen of combined cyclophosphamide and busulfan (40.74%), and cyclosporine associated with methotrexate was the most frequent prophylactic drug combination for GvHD (62.98%). Twenty-two patients (81.0%) were diagnosed with systemic cGvHD, and 17 (63.0%) with oral cGvHD (Table 2).

**Table 2:** Transplant data (n = 27).

**Variable**	**n**	**%**
**Initial Diagnosis**		
Acute lymphoblastic leukemia	12	44.4
Acute myeloblastic leukemia	7	25.9
Chronic myeloid leukemia	3	11.1
Myelodysplastic syndrome	2	7.4
Aplastic anemia	3	11.1
**Conditioning Regimen**		
Cyclophosphamide + Busulfan	11	40.7
Cyclophosphamide + TBI	9	33.3
Cyclophosphamide + Fludarabine	1	3.7
Cyclophosphamide + Busulfan + TBI	1	3.7
Busulfan + Melphalan	1	3.7
Busulfan + Fludarabine	4	14.9
**cGvHD Prophylaxis**		
CSA + MTX	17	63.0
CSA + MTX + ATG	4	14.8
CSA + MTX + MFM	1	3.7
CSA + Tacrolimus	1	3.7
CSA	1	3.7
CSA + ATG	1	3.7
CSA + MFM	1	3.7
MTX + ATG	1	3.7
**Donor Type**		
HLA-mismatched related	1	3.7
HLA-identical sibling	21	77.8
Missing	5	18.5
**Stem Cell Source**		
Bone marrow	20	74.1
Umbilical cord blood	1	3.7
Peripheral blood	6	22.2
**Chronic systemic GvHD**		
No	5	18.5
Yes	22	81.5
**Chronic oral GvHD**		
No	10	37.0
Yes	17	63.0

TBI: Total body irradiation CSA: Cyclosporine A; MTX: Methotrexate; ATG: Anti-thymocyte globulin; MFM: Mycophenolate mofetil; HLA: Human leukocyte antigen; GvHD: Graft-versus-host disease.

Sialometry performed five years post-transplant showed normal values in most patients (63.0%), with a median of 0.5 mL/min and median pH of 6.5. Xerostomia was reported by 33.3% of patients (Table 3).

**Table 3:** Sialometry, pH and xerostomia data five years post-transplant (n = 27).

**Variable**	
Sialometry (mL) 5 years post-transplant - median (IQR)	0.5 (0.2 - 0.8)
**Sialometry 5 years post-transplant (categorized)** – n (%)	
Asialia	1 (3.7)
Low	5 (18.5)
Very low	3 (11.1)
Normal	17 (63.0)
Missing	1 (3.7)
**Xerostomia 5 years post-transplant** – n (%)	
No	18 (66.7)
Yes	9 (33.3)
**Salivary pH** - (median (IQR)	6.5 (6.0 - 7.0)

No statistically significant association was found between sialometry five years after allogeneic HSCT and the occurrence of chronic systemic, oral, or combined GvHD (Table 4).

**Table 4:** Association between hyposalivation and oral cGvHD five years post-transplant (n = 27) n (%).

**GvHD**		**Asialia**	**Low**	**Very low**	**Normal**	**p**
		n = 1	n = 5	n = 3	n = 17	
Systemic GvHD	No	0 (0.0)	2 (40.0)	0 (0.0)	3 (17.6)	0.500
	Yes	1 (100.0)	3 (60.0)	3 (100.0)	14 (82.4)	
Oral GvHD	No	0 (0.0)	3 (60.0)	0 (0.0)	7 (41.2)	0.317
	Yes	1 (100.0)	2 (40.0)	3 (100.0)	10 (58.8)	
Systemic + Oral GvHD	No	0 (0.0)	2 (40.0)	0 (0.0)	2 (11.8)	0.358
	Yes	1 (100.0)	3 (60.0)	3 (100.0)	15 (88.2)	

GvHD: Graft-versus-host disease.

## Discussion

HSCT is a well-established treatment for various hematological malignancies, with favorable remission outcomes. However, it is frequently associated with complications, among which cGvHD is one of the most prevalent and challenging conditions to manage. Salivary gland dysfunction, including xerostomia and hyposalivation, is a common complication and can significantly impair quality of life and long-term outcomes [20].

This study included 27 patients, predominantly male (55.6%), with a median age of 38 years and a median of 64 months post-transplant. These demographics align with prior studies such as Piccin et al. [21], Bezinelli et al. [22], and de Paula Eduardo et al. [23], which also reported male predominance and a similar age distribution. The younger median age in this cohort is consistent with Bezinelli et al. [22] and Hayden et al. [24] (30 and 36 years, respectively), whereas Piccin et al. [21] reported a higher median of 47 years.

ALL was the most frequent underlying condition (44.4%), comparable to findings by Piccin et al. [21], who reported acute leukemias in 62.3% of cases. In contrast, Bezinelli et al. [22] and Woo et al. [25] found higher frequencies of lymphomas (27.3% and 39.0%, respectively), while Blijlevens et al. [26] reported a predominance of multiple myeloma (53.3%).

The majority of our patients received grafts from HLA-identical sibling donors, in agreement with Bezinelli et al. [22] and Boer et al. [27]. However, other studies such as Legert et al. [28], Cheon et al. [29], and Abasaeed et al. [1] reported greater use of matched unrelated donors.

Regarding conditioning regimens, 40.7% of patients received busulfan plus fludarabine, a combination also used in 53.8% of the cohort of Cheon et al. [29]. Fludarabine was employed in 49.1% of patients by Piccin et al. [20], whereas melphalan was more commonly used in the studies by Bezinelli et al. [22], Blijlevens et al. [26], and Wysocka-Słowik et al. [30], illustrating variability based on disease type and transplant protocols.

Prophylaxis for cGVHD most frequently included cyclosporine (Cyclosporine capsules) plus methotrexate (Methotrexate), consistent with Legert et al. (67.8%) [28] and Boer et al. (89.0%) [27].

In the present cohort, 63% of patients had normal unstimulated salivary flow approximately five years post-transplant, with a median flow rate of 0.5 mL/min. These results align with Bulthuis et al. [31], who reported a mean flow rate of 0.36 mL/min five years post-HSCT, yet found that 73% of patients still experienced oral dryness. Similarly, Piccin et al. [21] observed preserved salivary function despite high rates of xerostomia.

Xerostomia was reported by 33.3% of our patients, similar to Piccin et al. (22.6%) [21] and Bezinelli et al. (39%) [22], the latter noting a median symptom duration of ten days. Other studies report even higher rates among cGvHD patients, including Hiroki et al. (54.5%) [32], Nakamura et al. (61.1%) [33], and Coracin et al. (50% at Day +100) [34]. These findings are consistent with Bulthuis et al. [31], where 73% of survivors experienced chronic oral dryness, underscoring the persistence of xerostomia despite preserved flow rates.

The findings of this study reinforce a relevant dissociation between objective salivary flow and subjective xerostomia, suggesting that normal salivary flow rates do not exclude clinically relevant dry-mouth symptoms. This indicates that xerostomia may reflect additional factors beyond quantitative salivary output. Early identification and management are essential to mitigate these risks and enhance quality of life. Regular dental follow-up and individualized interventions remain critical, particularly for those with signs of glandular dysfunction.

Salivary flow was assessed only once, approximately five years post-HSCT, limiting insight into early functional changes. However, Wysocka-Słowik et al. [30] reported transient oral dryness in 89% and 92% of patients during the first- and second-weeks post-transplant, respectively. Abasaeed et al. [1] observed persistent xerostomia and thick saliva at Day +80, and Andersson et al. [35] noted symptoms one year after myeloablative conditioning. Coracin et al. [34] also reported xerostomia in 50% of patients at Day +100, while Cheon et al. [29] found a lower prevalence (22.4%) among survivors. These findings reinforce xerostomia as a frequent and long-lasting symptom with significant clinical relevance, even in the context of preserved salivary flow. It is important to note that even in cases in which patients have a normal salivary flow, but complain of xerostomia, it is necessary to implement treatments that provide comfort to the patient, because sialometry may be within normal parameters, but may be lower than what the patient had before HSCT. Subjective xerostomia despite preserved salivary flow may reflect qualitative alterations in saliva composition rather than quantitative dysfunction alone. Changes in salivary proteins, antimicrobial constituents, and antioxidant capacity after HSCT may compromise lubrication and salivary defense mechanisms, potentially contributing to dryness symptoms even when flow rates remain within normal limits. This suggests that compositional changes in saliva may represent an additional mechanism underlying xerostomia in HSCT survivors [8].

Bulthuis et al. [31] also observed that patients with persistent salivary dysfunction had higher dental treatment needs over five years, especially among those with reduced stimulated flow at 12 months. This highlights the importance of early and sustained monitoring.

Additionally, van Leeuwen et al. [8] described a dynamic post-HSCT salivary inflammatory profile, with initial elevations in albumin and pro-inflammatory cytokines, followed by normalization and a rise in antimicrobial proteins by six months potentially reflecting the functional recovery observed in the current cohort.

In the present study, the median salivary pH was 6.5, a value within the physiological range and consistent with a stable oral environment. Similar findings were reported by Bulthuis et al. [36], who observed that group mean values for unstimulated saliva pH fluctuated between 6.1 and 6.3 (overall individual range: 5.0–7.6), while stimulated saliva pH means ranged from 6.6 to 7.0 (overall individual range: 5.8–8.0) in patients undergoing HSCT. Complementarily, Arduino et al. [37] reported, in an adult Italian post-HSCT population, a mean salivary pH of 7.41 ± 0.61, with no statistically significant difference between patients with and without hyposalivation (7.38 ± 0.75). However, a significant reduction in pH was noted in individuals with erosive oral lesions (6.97 ± 0.58). The control group presented a mean pH of 7.28 ± 0.73, with no significant difference compared to the HSCT group. These findings suggest that, even in clinical conditions potentially associated with altered salivary function, salivary pH may remain within physiological parameters.

The limitations of this study are related to the relatively young age of participants (38 years), since salivary flow decreases with age. Furthermore, the inclusion of both adolescents and adults may have introduced confounding variables. The absence of data regarding variables such as antidepressant/anticholinergic, antihypertensive, and diuretic use, as well as uncontrolled diabetes, constitutes an important limitation, as these factors may have influenced the salivary flow assessment. This is particularly relevant given that tricyclic antidepressants have been associated with xerostomia and reduced salivary flow, with previous studies reporting up to a 58% reduction in stimulated salivary flow in medicated patients [38]. Likewise, antihypertensive agents, particularly β-adrenergic blockers, diuretics, and angiotensin-converting enzyme inhibitors (ACE), have been linked to salivary alterations, with higher xerostomia prevalence and lower salivary flow observed in medicated hypertensive patients compared with controls [39,40]. Collectively, these findings highlight the potential confounding effect of systemic medications on the interpretation of salivary function in HSCT survivors. The number of patients without a diagnosis of systemic cGvHD (n = 5) or oral cGvHD (n = 10) was too small to allow for robust statistical comparisons of patients presenting the condition. Consequently, the analyses were restricted to patients with cGvHD, focusing on the association between hyposalivation and the disease. The limited evaluation of the non-cGvHD group may have reduced the study’s power to detect significant intergroup differences. Nevertheless, all eligible patients within the established period and criteria were included to preserve cohort representativeness. Future studies with larger non-cGvHD cohorts are needed to more precisely determine hyposalivation prevalence in this population. These results are consistent with previous findings, particularly regarding the dissociation between salivary flow and xerostomia, reinforcing the importance of integrating both subjective and objective assessments into post-HSCT care protocols to optimize oral health outcomes.

## Conclusion

This study demonstrates that most patients maintain normal unstimulated salivary flow five years after HSCT, with no statistically significant association between hyposalivation and systemic or oral cGvHD. Nevertheless, a substantial proportion of patients continue to report xerostomia.

These findings highlight a clear dissociation between objective salivary flow and subjective oral dryness, underscoring the importance of incorporating both measures in the clinical evaluation of HSCT survivors. The persistence of xerostomia despite preserved salivary flow suggests that qualitative alterations in saliva may play an important role in symptom development.

Individualized management strategies are essential to address xerostomia and improve long-term oral health and quality of life in this population. Further studies are warranted to better elucidate the underlying mechanisms, particularly the role of conditioning regimens and cGvHD–related changes in salivary gland function.

## Conflicts of interest

None

## Data Availability

The data that support the findings of this study are available from the corresponding author upon reasonable request.
